# A population database analysis to describe the residual burden of varicella in Italy—a high vaccination coverage area—from 2004 to 2022

**DOI:** 10.3389/fpubh.2025.1412620

**Published:** 2025-03-03

**Authors:** Elisa Barbieri, Silvia Cocchio, Patrizia Furlan, Antonio Scamarcia, Luigi Cantarutti, Carlo Giaquinto, Vincenzo Baldo

**Affiliations:** ^1^Division of Paediatric Infectious Diseases, Department for Women’s and Children’s Health, University of Padua, Padua, Italy; ^2^Department of Cardiac Thoracic and Vascular Sciences and Public Health, University of Padua, Padua, Italy; ^3^Società Servizi Telematici – Pedianet, Padova, Italy

**Keywords:** varicella (chickenpox), children, Italy, epidemiology, disease complications

## Abstract

**Introduction:**

In Italy, universal varicella vaccination (VV) started in 2017 with a two-dose schedule administered in children aged 12–15 months and 5–6 years, achieving 90% coverage in 2019, though with regional variations. To address the limitations in surveillance databases for capturing varicella episodes, the study aimed to evaluate the burden of varicella disease in the pediatric population using a primary care database.

**Methods:**

This cohort study used data from Pedianet, a comprehensive database of 193 family pediatricians in Italy. The incidence rate (IR) of varicella (International Classification of Diseases, Ninth Revision, Clinical Modification [ICD-9-CM] codes 052 and 052.0–52.9) was evaluated in children aged below 15 years, from January 2004 to April 2022, categorized by calendar year and region. Subjects were followed up from 2004 or their enrollment date until the end of assistance or the study period. Comorbidities and complications were identified.

**Results:**

A total of 253,221 children aged below 15 years (resulting in a total follow-up of 1,430,249 person-years) were included in the study. A total of 35,614 varicella index cases were identified in 35,199 subjects (13.9%), with 1.2% experiencing two infections. Complications following varicella occurred in 467 children (1.3%), primarily affecting the skin and soft tissue (46.3%) and the respiratory tract (22.3%). The IR in regions that implemented the VV program before 2017 ranged from 38.3 per 1,000 person-years in 2007 to 0.8 per 1,000 person-years in 2022, while in those that implemented the VV in 2017, the IR decreased from 49.8 per 1,000 person-years in 2017 to 3.2 per 1,000 person-years in 2022. In the Veneto Region, following the implementation of VV in 2006, the IR significantly decreased by 20.5 annually (95% CI: −23.4, −17.5), ranging from 50.2 per 1,000 person-years in 2006 to 1.2 per 1,000 person-years in 2022.

**Conclusion:**

The implementation of VV drastically reduced the IR of varicella, further confirming the importance of universal VV coverage.

## 1 Introduction

Varicella zoster virus (VZV) is a highly contagious infection found worldwide, affecting between 2 and 16 individuals per 1,000 annually in the absence of preventive strategies or vaccination (1). VZV infection leads to two clinically distinct forms of the disease: varicella (chickenpox) and herpes zoster (shingles) (1).

Clinical manifestations of varicella generally develop after an incubation period of 10–21 days following exposure; they include a prodrome of fever, malaise, headache, anorexia, or pharyngitis, followed by a characteristic generalized pruritic vesicular rash. It is typically a mild-to-moderate disease in healthy children, in contrast to more severe cases in adults or immunocompromised patients of any age (1). Despite a benign, self-limited course in the majority of the episodes, severe complications can result after primary infection, leading to an increased risk of varicella-related hospitalization and mortality. The most frequent complications include skin and soft tissue infections, pneumonia, encephalitis, Reye’s syndrome, and hepatitis (1, 2).

Since 1995, the implementation of routine childhood immunization has significantly altered its epidemiology, leading to decreased disease incidence, complications, hospital admissions, and deaths among children and in the general population, thereby indicating strong herd immunity. In the United States, following the introduction of the varicella vaccination (VV) program, the mortality rate for varicella decreased from 0.41 to 0.05 per million (2).

Nevertheless, only 12 European countries have implemented universal VV programs (3). In Italy, the vaccination for varicella was adopted and offered in a universal program at different times in different regions [i.e., Sicily (2003), Veneto (2006), Apulia (2006), Tuscany (2008), Basilicata (2010), Calabria (2010), Sardinia (2011), and Friuli Venezia Giulia (2013)] before being offered at national level starting from 2017 in all the regions (4). Since 2017, the schedule adopted is a two-dose schedule in children aged 12–15 months and 5–6 years (5, 6).

In 2010, for those regions that implemented the universal varicella vaccination, the coverage rates increased by 80% (i.e., Sicily, Veneto, and Apulia) (7). In 2019, the national first-dose coverage for varicella reached 90.5% for the 2017 cohort, with stable rates in subsequent years, though regional variations were observed (6).

Epidemiological data show a drastic reduction in varicella incidence, with almost half of the episodes from 2003 to 2013 among some Italian regions, but no up-to-date data are available at the national level (6).

Due to limitations in electronic health record databases in capturing varicella episodes in Italy, we aimed to assess the burden of varicella disease in the pediatric population using a primary care database. This study aims to update epidemiological and clinical data on varicella disease in the Italian pediatric population, focusing on the trends of incidence, clinical presentation, risk factors, and possible complications over the past two decades.

## 2 Materials and methods

This population-based birth cohort analysis complied with the European Network of Centres for Pharmacoepidemiology and Pharmacovigilance methodological standards and did not require approval from the ethics committee.

### 2.1 Data source

The Pedianet database was used as the source of the study. Pedianet is a national population database that contains anonymous patient-level data of more than 500,000 children since 2004, corresponding to approximately 4% of the annual pediatric population, who received healthcare from 193 family pediatricians (FPs) from 1 January 2004 to 30 April 2022 in Italy who were part of the Pedianet network^1^ using the same software package (Junior Bit^®^, Padova, Italy) in their professional practice. The data were extracted for this study on 23 October 2022.

According to the Italian National Health Service, each child is assigned to an FP, who is the primary referral for health-related matters. In Italy, there is a tax-funded public healthcare system that provides universal access, and patients do not incur any direct costs related to primary care visits. The Pedianet database captures various types of patient-level information, including the reason for accessing healthcare, health status, demographic data, diagnosis and clinical symptoms (in a free text or ICD-9-CM codes), drugs (Anatomical Therapeutic Chemical Classification codes), specialist appointments, diagnostic procedures, hospital or emergency room (ER) admissions, vaccinations, growth parameters, and clinical outcome data. Data are anonymized with a monthly update to a centralized database based in So.Se.Pe.—the legal owner of Pedianet, in Padova. Informed consent from children’s parents is required to enter the data into the database. The data collected from the child’s parents or tutors by pediatricians is entered into a dedicated cloud that is already encrypted and anonymized. Pedianet researchers are unaware of the anonymization process and cannot identify the owner of the data in any way.

### 2.2 Study design and study population

This is a retrospective population-based birth cohort analysis aiming at assessing the variation in the incidence of varicella over calendar years between 2004 and 2022 in children aged below 15 years. Any individual with missing data on age or sex was excluded because they were considered missing at random.

The varicella diagnoses were collected either with ICD-9 CM codes (052 and 052.0–52.9) or as free text in the medical notes (in Italian “varicella”) and manually validated by a clinical data manager to exclude possible false positives. Each visit, treatment and healthcare resource registered within 30 days from the varicella incident date were considered part of the same episode.

Regions were grouped in Veneto (where the universal VV program started in 2006), regions that introduced VV before 2017, named “Regions Pre2017” (Friuli Venezia Giulia, Sicilia, Puglia, Sardegna, and Toscana), and regions that offered the universal VV program starting from 2017, named “Regions 2017.”

Complications were associated with the varicella episode if a diagnosis of pneumonia, skin and soft tissue infection, neurological complications, and hepatitis were recorded as outpatient or inpatient visits within 30 days from the varicella episode index date. Every death event (not categorized as accidental death or suicide) was retrieved from the clinical diary and diagnosis field that were associated with the varicella episode if the event was recorded within 30 days from the varicella episode index date.

### 2.3 Statistical analysis

Descriptive analyses were summarized through tabulation and graphical representation of the means, medians, and standard deviations for continuous variables, as well as frequency distributions for categorical variables.

Each subject was followed from the 15th day of birth or the enrollment date, within 6 months of birth, until the end of assistance or the first varicella diagnosis or to the 30 April 2022, whichever came first. The incidence rate (IR) of varicella episodes was calculated by dividing the number of varicella episodes during the follow-up period by the total person-time computed as the number of years elapsed between the start and the end of the follow-up, expressed per 1,000 person-years. Furthermore, 95% confidence intervals were calculated by a group of regions. Significant trends over the years considered were assessed as average annual percent changes (AAPC) using the joint-point regression (8). The variation in the prescription rate was evaluated using the chi-square test. A *p*-value below 0.05 was accepted as statistically significant. Statistical analyses were performed using the Statistical Package for the Social Sciences (SPSS, IBM, Armonk, NY, USA) version 28.0 software program.

## 3 Results

**Figure 1:**
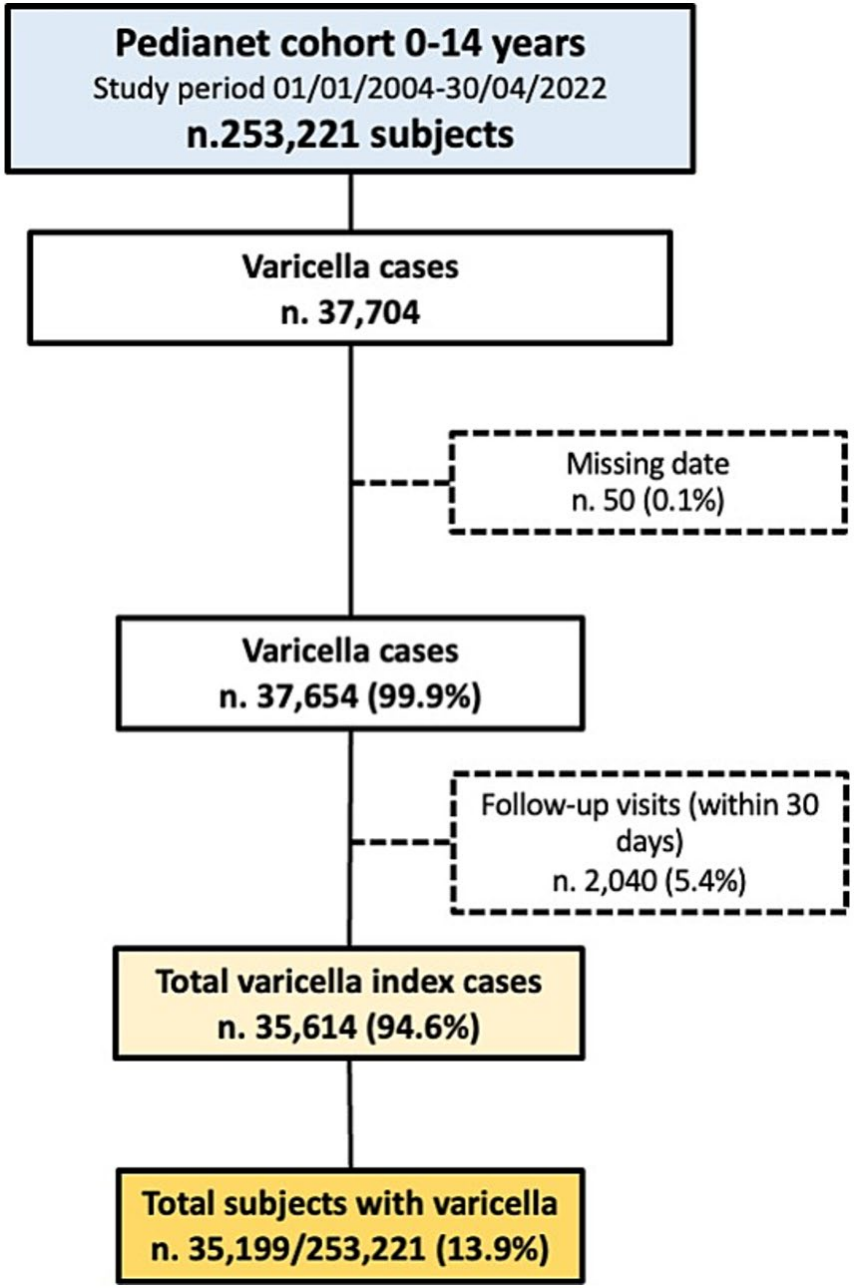
Flowchart of varicella index cases selection. Pedianet, 2004–2022.

During the 19-year-study period, 253,221 children aged below 15 years followed for a total of 1,430,249 years (mean follow-up of 5.6 years per person) were included in the analysis. A total of 35,614 varicella index cases were retrieved in 35,199 subjects (13.9%) (Figure 1). Overall, 51.7% were men, and Veneto was the region most represented accounting for 41.2% of total subjects. A total of 7,291 (2.9%) of children had at least a comorbidity; prematurity was the most frequent (1.3%, Supplementary Table 1s), followed by asthma (0.8% Supplementary Table 1s).

In 79.2% of children, the first varicella case was recorded after the age of 2 years (Table 1). A total of 415 children (1.2%) had two infections, and the majority of the cases (95.4%) occurred within 6 years of the index case. A total of 467 children (1.3%) had at least one varicella episode with at least a complication, the majority of which related to the skin and soft tissue (46.3%) and the respiratory tract (22.3%) (Table 1).

**Table 1:** Description of varicella cases by age class, number of varicella infections, and complications in children aged 0–14 years in Italy.

	Subjects with varicella (*N* = 35,199)
	*N* (%)
Age group at first varicella episode	
<1 year	3,151 (9.0)
1 year	4,172 (11.9)
2–5 years	22,681 (64.4)
>6 years	5,195 (14.8)
Number of varicella infections per child	
1	34,784 (98.8)
2	415 (1.2)
Complications	
No	34,732 (98.7)
Yes	467 (1.3)
Skin/mucosal/soft tissue	216 (46.3)
Respiratory	104 (22.3)
Neurological	11 (2.4)
Not specified	136 (29.1)

Pedianet, 2004–2022.

Overall, from 2004 to 2022, the incidence rate of varicella was 24.61 cases per 1,000 person-years, and no difference by sex was noted. The IR differed among the 13 regions, ranging from a minimum of 1.6 and 2.1 varicella episodes per 1,000 person-years in Sicily and Puglia regions, respectively, to a maximum of 61.7 varicella episodes per 1,000 person-years in the Marche region (Table 2).

**Table 2:** Demographic characteristics, varicella episodes, and varicella incidence rates (with 95% CI) in children aged 0–14 years in Italy.

	Total subjects of the cohort *N* (%)	Total subjects with varicella *N* (%)	Follow-up (years)	Incidence × 1,000 person-years (95% CI)
Total	253,221 (100)	35,199 (13.9)	1,430,249	24.61 (24.36–24.86)
Sex				
Males	131,009 (51.7)	18,327 (14)	740,937	24.73 (24.38–25.09)
Females	122,212 (48.3)	16,872 (13.8)	689,312	24.48 (24.11–24.84)
Region				
Abruzzo	17,943 (7.1)	5,077 (28.3)	92,987	54.6 (53.14–56.06)
Campania	20,371 (8)	2,666 (13.1)	113,402	23.51 (22.63–24.39)
Friuli Venezia Giulia	5,869 (2.3)	1,631 (27.8)	30,111	54.17 (51.61–56.72)
Lazio	8,492 (3.4)	1,638 (19.3)	43,626	37.55 (35.76–39.33)
Liguria	1,429 (0.6)	321 (22.5)	6,788	47.29 (42.24–52.34)
Lombardia	17,455 (6.9)	3,995 (22.9)	73,733	54.18 (52.55–55.82)
Marche	22,306 (8.8)	6,712 (30.1)	108,857	61.66 (60.23–63.09)
Piemonte	18,783 (7.4)	4,924 (26.2)	91,426	53.86 (52.39–55.32)
Puglia	2,578 (1)	41 (1.6)	19,732	2.08 (1.44–2.71)
Sardegna	6,089 (2.4)	874 (14.4)	34,010	25.7 (24.02–27.38)
Sicilia	22,543 (8.9)	234 (1)	143,869	1.63 (1.42–1.83)
Toscana	4,973 (2)	444 (8.9)	30,076	14.76 (13.40–16.13)
Veneto	104,390 (41.2)	6,642 (6.4)	641,631	10.35 (10.10–10.60)

Pedianet, 2004–2022.

**Figure 2:**
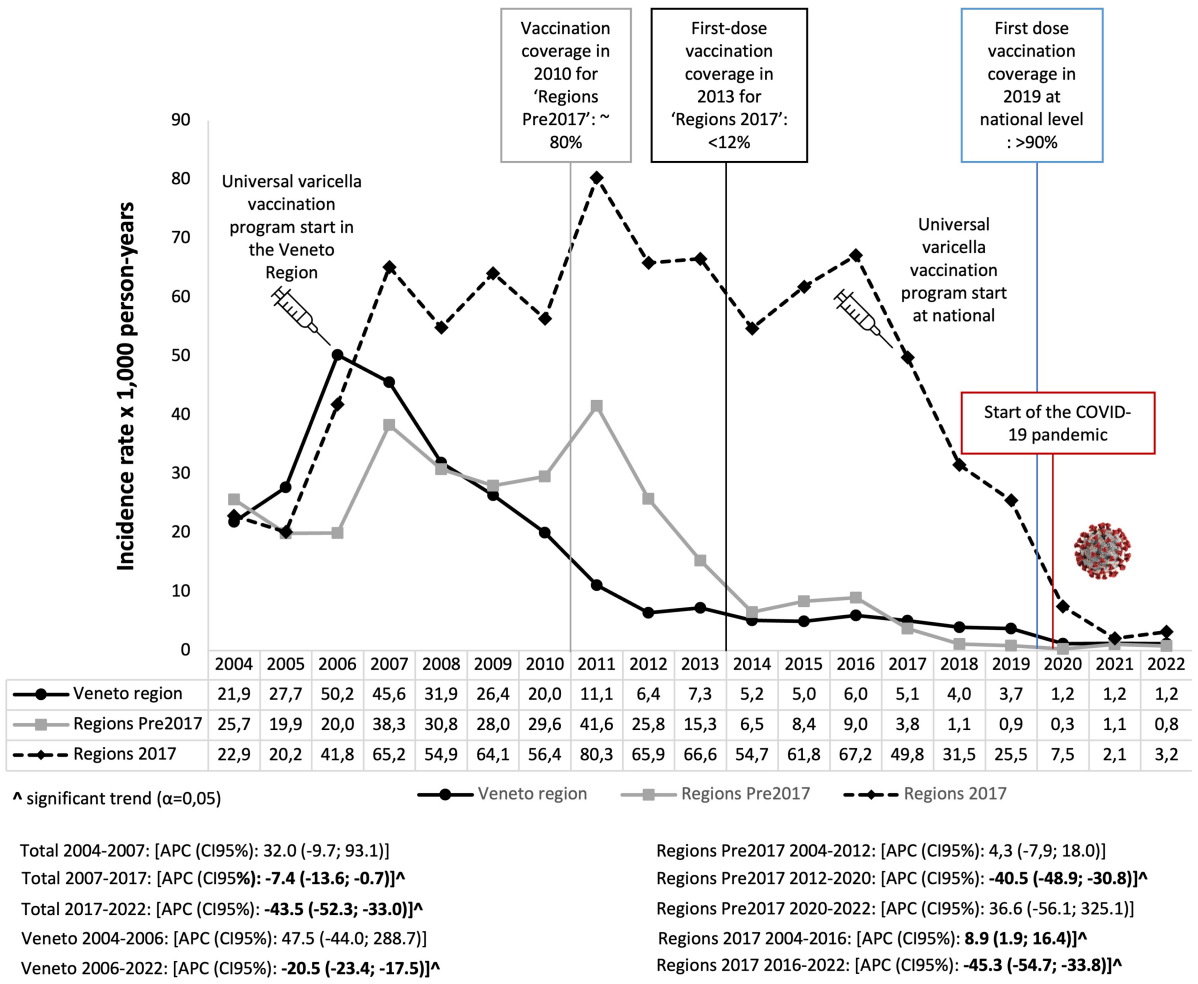
Annual trend of varicella expressed as the incidence rate of varicella episodes per 1,000 person-years in children aged 0–14 years in Italy. Pedianet, 2004–2022.

The IR varied from 52.6 episodes in 2007 to 1.8 per 1,000 person-years in 2022, with an annual percent change of −7.4 (95% CI: −13.6, −0.7) from 2007 to 2017 and of −43.5 (95% CI: −52.3; −33.0) from 2017 to 2022. Specifically, in the Veneto Region following the implementation of VV in 2006 (9), the IR significantly decreased annually by 20.5 (95% CI: −23.4; −17.5), ranging from 50.2 in 2006 to 1.2 per 1,000 person-years in 2022 (Figure 2). In regions where universal VV programs started before 2017 (Regions Pre-2017), the IR significantly decreased annually by 40.5 (95% CI: −48.9; −30.8), ranging from 25.8 in 2012 to 0.3 per 1,000 person-years in 2020 (Figure 2). For the regions that implemented VV programs in 2017, a significantly decreasing trend after the vaccination introduction was observed; the IR decreased by 45.3 (95% CI: −54.7, −33.8) (Figure 2).

## 4 Discussion

Continuous evaluation of the burden of vaccine-preventable disease, such as VZV, is necessary to promptly intervene in case of an increased trend in the incidence of the disease to inform further research to monitor the mutations in VZV genome, to discriminate vaccine virus from wild type virus, to study the phylogeny of VZV strains throughout the world, and to understand the evolution of the different clades of VZV. The implementation of VV drastically reduced varicella cases in all Italian regions, further confirming the importance of universal implementation of VV. In line with previous studies, the majority of the children were older than 2 years of age at first infection.

The incidence rates were very low in two Southern regions (Puglia and Sicilia), but the underreporting of cases was already noted (10). Children with comorbidities had lower incidence rates of varicella compared to the overall population, possibly because those considered at risk were more prone to adopt non-pharmaceutical measures to reduce the risk of catching an infection.

Our results align with what was previously published on the population in Italy. In particular, an analysis using surveillance data from 2001 to 2010 in Italy revealed an average annual varicella IR of 9.49 cases per 1,000 inhabitants in children aged below 14 years (88.8% of the cases in the overall population). Notably, in this period, the universal vaccination for varicella had already been adopted by different regions: Sicily (in 2003), Veneto (in 2006), Apulia (in 2006), and Tuscany (in 2008), and vaccination coverage rates increased up to 80% in 2010 (5).

Consequently, in Sicily, the incidence of varicella decreased from 105.7 in 2003 to 9.2 per 100,000 inhabitants in 2010; in Veneto, the incidence decreased from 225.5 in 2007 to 55.7 per 100,000 inhabitants in 2010; in Apulia, the incidence decreased from 121.7 in 2006 to 13.1 per 100,000 inhabitants in 2010 (4), while in Tuscany, the incidence decreased from 350 in 1994 to 20 cases per 100,000 inhabitants in 2018 (11).

It should be considered that the sharp decline in the incidence rates from 2019 to 2022 might be attributed to the COVID-19 lockdown to some extent. Indeed, In Italy, voluntary and mandatory non-pharmaceutical interventions (NPIs) to reduce severe acute respiratory syndrome coronavirus 2 (SARS-CoV-2) circulation were first implemented in February 2020 and remained until the 18 May 2020 and included school (including nursery) and university closures, intrapersonal distancing of at least 1 m, ban of travel outside the city in which one person resides for reason other than work, public places and store closures, suspension of all public and private events including religious ceremonies (12). Notably, a surveillance analysis from France reported a reduction from 50 to 90% in varicella incidence from March through December 2020 (13). However, there was no active universal VV program in France during the study period (14).

The strength of this study is its size, generalizability, and representative coverage of pediatric patients. In Italy, enrolling in primary care is mandatory, and children are assigned to their FP based on their home proximity to the FP ambulatory.

A limitation of this study lies in its retrospective nature. The impossibility of confirming clinical assessment is a well-recognized limitation in working with real-world data because it may be subjective to the attending clinician. However, varicella is a well-defined disease typical of the pediatric age. Second, we could not assess the subjects’ specific VV information for this study. Hence, variations that were noted in the disease burden in children with comorbidities might have resulted from a higher vaccination coverage in more fragile children. Third, the first available data regarding varicella vaccination coverage at the national level from the Ministry of Health are for children aged 5–6 years in 2013 (2006 birth cohort) (15). Hence it was not possible to fully assess the trend variation for the whole study period based on coverage rates and possible effect of regional catch-up programs. Fourth, limited representativeness in some regions was already reported with this database; hence. The regional estimates should be evaluated with cautiousness.

## Data Availability

The data analyzed in this study is subject to the following licenses/restrictions: the data used in this study cannot be made publicly available due to Italian data protection laws. The anonymized datasets generated during and/or analyzed during the current study can be provided on reasonable request, from the corresponding author, after written approval by the Internal Scientific Committee (info@pedianet.it). Requests to access these datasets should be directed to Internal Scientific Committee (info@pedianet.it).

## Footnotes

^1^ www.pedianet.it
