# Supplementary material for: A population database analysis to describe the residual burden of varicella in Italy—a high vaccination coverage area—from 2004 to 2022
Source: Front Public Health. 2025 Mar 3;13:1412620. doi: 10.3389/fpubh.2025.1412620 (PMC11911323; doi:10.3389/fpubh.2025.1412620)
Supplement: Supplementary file 1 [file Table_1.docx]

**Supplementary**

**A POPULATION DATABASE ANALYSIS TO DESCRIBE THE RESIDUAL BURDEN OF VARICELLA IN ITALY, A HIGH VACCINATION COVERAGE AREA, FROM 2004 TO 2022**

**Table 1s** –Clinical comorbidities, varicella episodes and varicella incidence rates (with 95% CI) in children 0-14 years of age in Italy. Pedianet, 2004-2022

|  | **Total subjects of the cohort N(%)** | **Total subjects with varicella N(%)** | **Follow-up (years)** | **Incidence x 1000 person-years (95%CI)** |
| --- | --- | --- | --- | --- |
| **Comorbidity** |  |  |  |  |
| Prematurity | 3216 (1.3) | 51 (1.6) | 13559 | 3.76 (2.73-4.79) |
| Asthma | 1984 (0.8) | 252 (12.7) | 19892 | 12.67 (11.11-14.22) |
| Other respiratory diseases | 42 (0) | 0 (0) | 267 | -- -- |
| Cardiovascular diseases | 513 (0.2) | 30 (5.8) | 3681 | 8.15 (5.25-11.05) |
| Neurological diseases | 812 (0.3) | 74 (9.1) | 6753 | 10.96 (8.47-13.44) |
| Metabolic diseases | 378 (0.1) | 43 (11.4) | 3647 | 11.79 (8.29-15.29) |
| Endocrine diseases | 402 (0.2) | 40 (10) | 3735 | 10.71 (7.41-14.01) |
| Cancers | 147 (0.1) | 13 (8.8) | 1253 | 10.38 (4.76-15.99) |
| Hepatic diseases | 19 (0) | 0 (0) | 147 | -- -- |
| Gastroenterological diseases | 36 (0) | 8 (22.2) | 274 | 29.18 (9.26-49.1) |
| Immunodeficiency | 2 (0) | 0 (0) | 12 | -- -- |
| Autoimmune diseases | 1 (0) | 0 (0) | 10 | -- -- |
| Blood diseases | 7 (0) | 1 (14.3) | 49 | 20.35 -- |
| Renal diseases | 23 (0) | 1 (4.3) | 170 | 5.88 -- |
| Organ transplant | 20 (0) | 2 (10) | 175 | 11.41 -- |
| Osteoarticular diseases | 5 (0) | 1 (20) | 52 | 19.16 -- |
| Multimorbidity | 70 (0) | 3 (4.3) | 458 | 6.55 -- |
